# Supplementary figures and images for: Family-led post-ICU discharge intervention for tracheostomized patients in India: Feasibility and formative impact evaluation
Source: PLoS One. 2026 May 29;21(5):e0348345. doi: 10.1371/journal.pone.0348345 (PMC13221049; doi:10.1371/journal.pone.0348345)

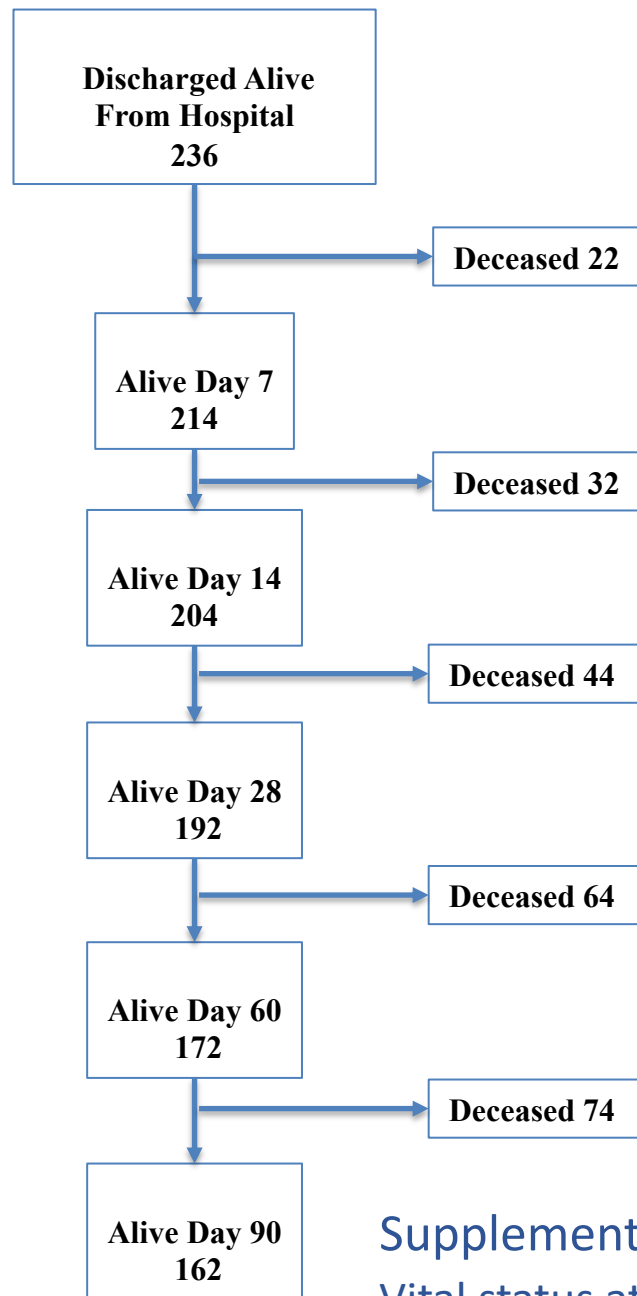

**Supplement Figure 1**  
Vital status at hospital discharge and follow-up

Supplement: S1 Fig — The figure reflects adherence to the training component of the intervention and reasons for non-compliance. (PDF) [file pone.0348345.s001.pdf]

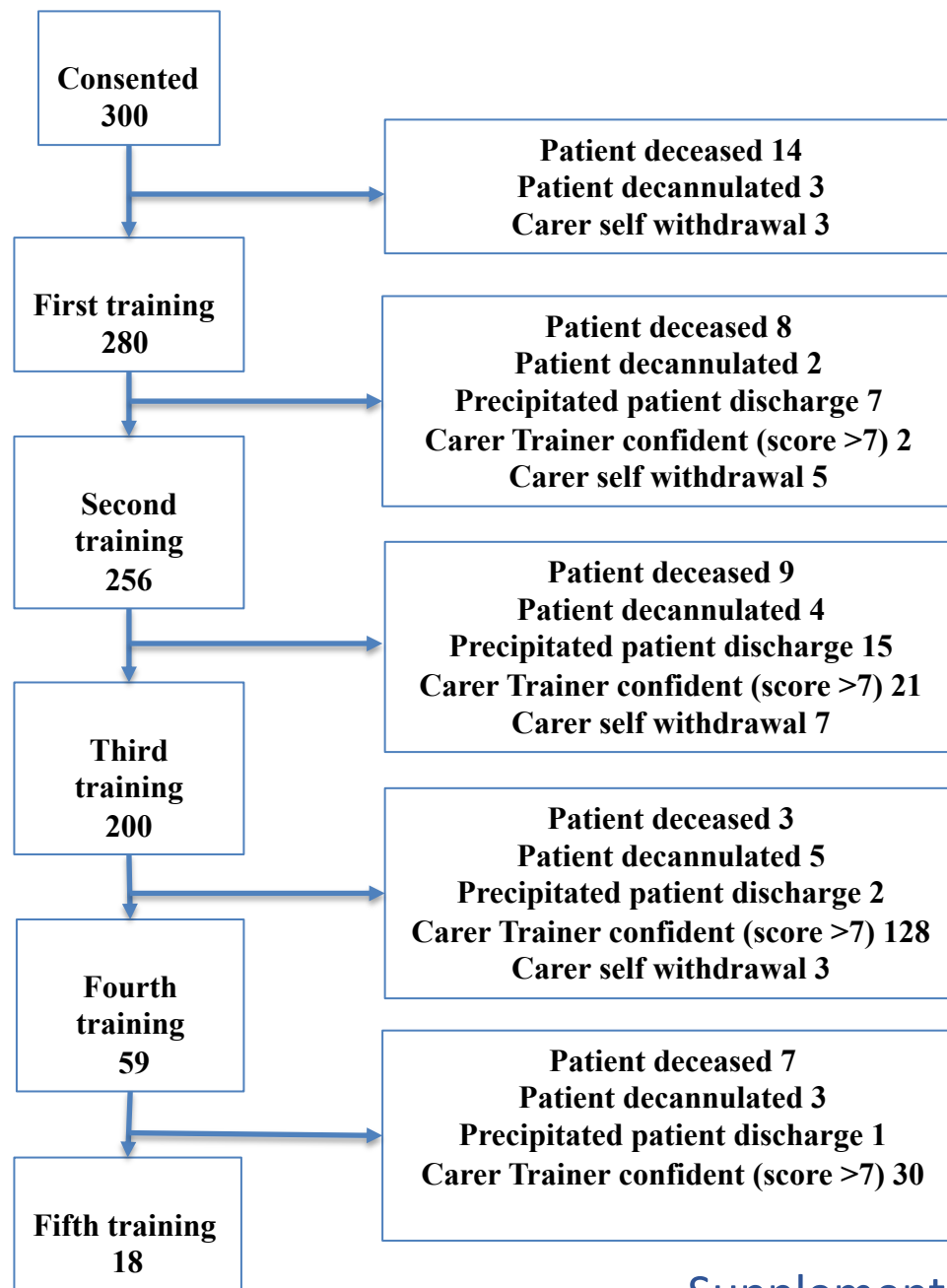

**Supplement Figure 2**

**Carer participation in training sessions**

Supplement: S2 Fig — (PDF) [file pone.0348345.s002.pdf]
