# Supplementary material for: Family-led post-ICU discharge intervention for tracheostomized patients in India: Feasibility and formative impact evaluation
Source: PLoS One. 2026 May 29;21(5):e0348345. doi: 10.1371/journal.pone.0348345 (PMC13221049; doi:10.1371/journal.pone.0348345)
Supplement: S1 Table — (DOCX) [file pone.0348345.s003.docx]

# Supplementary Table 3. Adaptations to Study and Intervention Design by Component Category

| Component Category | Study Component | Original Protocol | Reasons for Tailoring and/or Adaptations | Modified Protocol |
| --- | --- | --- | --- | --- |
| Screening and Counselling | Inclusion Criteria | i. Expected to survive to discharge and to need tracheostomy beyond ICU discharge. Hemodynamically stable, on weaning mode of ventilation. ii. Within a radius of 200 km. | Pressure on ICU beds required early consenting and training. Worries about equipment safety and logistics were resolved. | All tracheostomy patients are expected to survive, even on ventilator/vasopressors. Any patient-carer dyad accepting the intervention (equipment transport charged beyond 200 km). |
| Hands-on Carer Training | Hands-on Training of Carer | One-on-one training. One family at a time. Minimum 3 sessions. | Carers with learning difficulties benefited from observing other carers. | Invited struggling carers to group sessions or advanced-stage carers’ sessions. |
| mHealth app | Videos of Common Procedures | Videos filmed in simulated settings. | Carers expressed a preference for more realistic content. | Videos for suctioning, nasogastric feeding, and bathing were refilmed in real-world settings. |
| Equipment Bank | Information leaflet  Eligibility for Free Equipment | ‘Free access to equipment bank for patient care’.  Only patients below the poverty line (BPL status) could access the free equipment bank. | ‘Free’ label led to misuse and lack of engagement in training.  Providing initial support improved acceptance. | Emphasised that free equipment is conditional on passing a knowledge test and demonstrating basic caregiving skills.  One month of free access for all; more extended support is considered on a case-by-case basis. Clear explanation that support is temporary and conditional. |
| Post-Discharge Follow-up | Exclusion Criteria | i. Bedbound, but decannulated pre-discharge, were excluded. ii. Carers not trained adequately due to late consent or a conflicted situation. | Rapport continued post-discharge. Conflict/change in carers was common; stakeholders agreed tech could help. | i. Patients needing enteral feeding/catheter care were included. ii. Carers willing to use the mobile app were included. |
