# Supplementary material for: Family-led post-ICU discharge intervention for tracheostomized patients in India: Feasibility and formative impact evaluation
Source: PLoS One. 2026 May 29;21(5):e0348345. doi: 10.1371/journal.pone.0348345 (PMC13221049; doi:10.1371/journal.pone.0348345)
Supplement: S2 Table — (DOCX) [file pone.0348345.s004.docx]

# Supplementary Table 4.

| **Outcome** | **Instrument** | **Timepoint** |
| --- | --- | --- |
| Intervention acceptability | AIM | Year 1 dyads |
| Intervention appropriateness | IAM | Year 1 dyads |
| Intervention feasibility | FIM | Year 1 dyads |
| Carer confidence | Likert 0–10 | After each training session |
| Training assessment | Likert 0-10 | After carer confidence Score >6 in each module |
| Patient QoL | EQ-5D | Discharge, Day 14, Day 28 |
| Caregiver burden | CBS | Discharge, Day 14, Day 28 |
| Clinical outcomes | Survival, discharge timing | Discharge, Day 14, Day 28 |
| Interviews, Reflexive Meetings | Guide | Throughout the implementation |

## Outcome variables with corresponding assessment time points
