# Supplementary material for: Family-led post-ICU discharge intervention for tracheostomized patients in India: Feasibility and formative impact evaluation
Source: PLoS One. 2026 May 29;21(5):e0348345. doi: 10.1371/journal.pone.0348345 (PMC13221049; doi:10.1371/journal.pone.0348345)




**Table of Contents**

**Table of Contents**

[***1.0 STUDY SUMMARY 2***](#_46sp3px0hw29)

[***2.0 SCIENTIFIC ABSTRACT 3***](#_qeyd5459pn2r)

[***3.0 LAY SUMMARY OF RESEARCH 6***](#_lb2x42frr3ek)

[***4.0 BACKGROUND AND RATIONALE 7***](#_7ye9dwyhx9wu)

[***5.0 STUDY DESIGN 8***](#_dd767tcxwrgd)

[***6.0 PARTICIPANT ELIGIBILITY CRITERIA 13***](#_fvdyq8l2yqy6)

[***7.0 STUDY LOCATION 14***](#_z1oyjdcu06sx)

[***8.0 DURATION OF ENROLLMENT AND FOLLOW-UP 14***](#_p5shyzpuapqn)

[***9.0 DESCRIPTION OF INTERVENTION AND ADMINISTRATION 14***](#_ct772aatlanc)

[***10. DATA COLLECTION TIME POINTS 15***](#_bm68pr391ev9)

[***11. AMENDMENTS TO INITIAL PLAN AND PROTOCOL WITHIN THE STUDY PERIOD 17***](#_a6ovmmh56zhg)

[***12.0 REFERENCES 19***](#_dszj9mfwdim7)

# 1.0 STUDY SUMMARY

| **Study Title** | Evaluation of AIIMS ICU Rehabilitation (AIRe): a home care intervention facilitating family-led discharge of chronic critically ill patients with a tracheostomy in India. |
| --- | --- |
| **Short Title** | Is the AIR family-led home rehabilitation intervention effective in enabling timely hospital discharge? |
| **Study Acronym** | AIR MCT |
| **Study Design** | Pragmatic multi-centre, quasi-experimental study with an interrupted time series analysis including a control arm. (Workstream 1) A process evaluation (Workstream 2) and qualitative exploration of patient-carer experiences (Workstream 3) are embedded in this study. |
| **Eligibility** | **Inclusion criteria**   1. Adult tracheostomised patients aged>18 in Neurology or Neurosurgery ICUs 2. Expected to survive, and need tracheostomy for more than 2 weeks after being declared 'Ready For Step Down- RFSD) from the ICU by the treating physician/ surgeon. 3. Availability of a caregiver who consent-family-ledly led home discharge.   **Exclusion criteria**   1. Patients without an identified family who are carer-willing for home care, 2. Patients needing greater organisational support than is manageable at home (home ventilation excluded) 3. Current participation in an interventional trial with conflicting therapies or primary outcomes |
|  |  |
| **Recruitment sample size** | Primary analysis will be a pre-post analysis. Secondary analysis will be at sixteen time points (at weekly intervals) before and 16 time points after the intervention. Approximately 5 discharges at every time point. (<2 discharged patients per centre per week)  Intervention arm: Approx. 160 patients over 10 months.  Control arm: Similar. |
| **Study duration** | 36 months |
| **Primary Outcome Measure** | Time to Discharge- Defined as days from the clinician declaring Ready for Step Down (RFSD) to home discharge. |
| **Secondary**  **Outcome**  **Measures** | 1. Patient and Carer Reported Outcomes measured at 30 days. 2. Health-related QoL (EQ-5D-3L) of patients at hospital discharge and at 30 days 3. Carer Burden Scores of carers at hospital discharge and at 30-day post-discharge 4. Measures of safety   a) Hospital device-associated infections, e.g., CAUTI, VAP, HAP, in the rate of events within the group per unit time.  b) Clinical outcomes – Bed sores, Tracheostomy block, Clinical Deterioration, readmission, and mortality (rate/ unit time).   1. Measures of process 2. Adherence to the intervention  - % consented - % Trained for at least three sessions - Number of sessions missed. - Subjective and objective scores of carers' confidence post-training - % using the mHealth app at least once spontaneously after home discharge - % 1 week home visits completed - Data entry (completion)  1. Clinical 2. Measures for Direct Analysis 3. Resource use and expenditure questionnaire |

# 2.0 SCIENTIFIC ABSTRACT

**Background** Tracheostomy, a procedure to create an opening in front of the trachea when performed on critically ill patients in intensive care units (ICUs), decreases the duration of mechanical ventilation, incidence of nosocomial infections and length of ICU stay. Its use early during (1) neuro-ICU stay is associated with improved chances of early rehabilitation and discharge due to decreased secondary insults and hospital-acquired morbidity. (1) However, when there is a lack of out-of-hospital state-sponsored or low-cost rehabilitation facilities for these chronically ill bedridden patients, timely discharge from the ICU and hospital becomes a challenge. These patients need higher intensity care at home, including care of the tracheostomy and indwelling catheters, home ventilation, nasogastric feeding, physiotherapy, and general nursing care. Fear, stigma and unpreparedness among family members and healthcare workers of caring for a patient with 'a hole in the neck' prolongs ICU and hospital stay, increasing the very morbidities the tracheostomy sought to avoid. Costs of care to the patient and family, as well as the health care system, increase as resources cannot be allocated to acutely ill patients.

To address this gap, at our centre in Eastern India, we have developed and pilot-tested the All-India Institute of Medical Sciences Intensive Care Unit (AIIMS ICU) Rehabilitation Intervention. The intervention, with its components of counselling, hands-on training, and education, facilitated discharge and post-discharge support, aims to implement family-led home rehabilitation of chronically critically ill tracheostomy patients. Adaptations were informed by a realist review of international literature on the barriers and facilitators to implementation of similar interventions and based on the MRC guidelines for evaluation of complex healthcare interventions. This paper presents a protocol for the effectiveness and process evaluation using a pragmatic design, including interrupted time series assessments in both intervention and comparison groups across three ICUs in India. (2)

**Aim**

To evaluate the AIIMS ICU Rehabilitation (AIR) intervention (effectiveness and process)

**Objectives**

- 1. To evaluate the impact of the AIR intervention on the timeliness of discharge from the hospital for chronically ill tracheostomised patients in 3 ICUs in India.
  2. To identify organisational and health system factors affecting the implementation of the intervention (process evaluation).
  3. To explore the family and healthcare provider experience of the intervention.

**Methods**

**Design Objective** 1.1 will use Interrupted time series analysis (ITS), enabling comparison over time of the population. (Workflow 1) The ITS design has been selected, given that we will have control over when the intervention is implemented in the sites. This will enable us to overcome potential challenges of between-group differences ICUs patients and providers), and potentially reduce the need to control for complex confounders (clinician behaviours, care provision/costing structures).

Objectives 1.2 and 1.3 will use mixed methods approach for the process evaluation.

**Interventions and duration.** At each site, we will compare the time to discharge of CCI tracheostomy patients, their impact on patient-family dyads, before (4 months) and after implementation of AIR (4 months). Each patient-carer dyad will be followed up after recruitment, up to one month after discharge from the hospital. Objectives 1.2 & 1.3 will proceed concurrently.

**Sample size and population.** Being an interrupted time series design, with the outcome being the mean time to discharge from the ICU/ Hospital for all eligible patients every week, there will be 16 time points before and 16 after the intervention that will provide trends of change after the intervention is implemented. To account for threats to internal validity, such as history, maturation, and instrumentation, we will employ a control group in the study. The control group will comprise non-tracheostomised patients admitted to the ICUs during the same weeks as the patients in the intervention group, the observation of interest being the mean time to discharge. There are estimated to be approximately 160 patients in the intervention arm and a similar number in the control arm. In the event that the expected number of recruitments per unit of time is inadequate, and data do not lend themselves to a stable ITS analysis, a pre-post analysis will be done, with the control arm in place.

**Primary outcome measure:** Time to discharge from ICU or hospital to home after the treating physician's assessment as 'stable' and suitable for home care.

**Secondary outcome measures:** Additional patient-reported outcomes, safety and process measures, and measures of cost to patient-carer.

**Data collection and Analysis.** Data will be collected before and after the implementation of the intervention to assess its effect. A Statistical Analysis Plan will be drawn up prior to any analysis and reviewed by the Data Safety Monitoring Committee.

**Timelines** Months 0-8: approvals and set-up; Months 8-18: process evaluation; Month 6-8: 3 units initiated; Month 8-18: all three units open (8-11 baseline data collection; 12 & 13 implementation of intervention; 14-18 post implementation data collection); Months 18-36: Patient follow up, Qualitative data collection, data cleaning, database lock, statistical cleaning, and dissemination of results.

# 3.0 LAY SUMMARY OF RESEARCH

Tracheostomy is a lifesaving procedure for patients with neurologic (brain) damage. Despite studies showing that it provides benefits such as decreased infection and time on ventilator and ICU, patients with tracheostomies and their family members (carers) suffer from a severe shortage of affordable rehabilitation facilities for these patients once they are ready to be discharged from the ICU and hospital because of various reasons: fear of caring for a patient with a 'hole -in -the neck', inadequate guidance and training of family members prior to discharge, lack of post discharge follow up. The fact is that these patients end up staying longer at hospitals, getting repeated infections leading to ICU readmissions- increasing the cost of care for patient families and leaving medical teams struggling to provide care to newer patients who need more acute attention.

The AIR intervention, however, aims to enable family-led home-based rehabilitation of these bedridden tracheostomy patients and offers patients and families something different.

i. The opportunity to discuss with counsellors and doctors about caring for their patients at home,

ii. Receive hand-held bedside training from trained nurses using modules (for tracheostomy care, indwelling catheter care, nasogastric feeding, back care, suctioning, home ventilation management and troubleshooting) that have been codeveloped by patient-family and healthcare workers, till they self-report confidence in their caring ability.

iii. Having access to free equipment for the first month of at-home rehabilitation.

iv. Facilitated discharge and follow-up for one month- ability to communicate with the rehabilitation team via a bespoke mHealth app that also acts as an education tool with a repository of videos and answers to frequently asked questions.

A preliminary study of the AIR intervention at one centre has shown that it is feasible and acceptable to patients, families, and healthcare workers. Families feel safe, confident, and happy with the AIR- many see faster recovery in their wards, and other carers can return to work faster, to begin daily earnings. However, our understanding of how much AIR can hasten discharge, decrease infections, and reduce costs for different varieties of patients in different settings is inadequate. The only way to know for sure is to compare longer periods with and without intervention in different hospitals. International trials do not faithfully explore issues that matter to bedridden tracheostomised patient- families, making it hard to assess the impact of home discharge and home care on real-life. To ensure that we capture these elements, we hope to liaise closely with all stakeholders in this study to better understand for whom, in which circumstances and how home rehabilitation intervention may or may not be effective.

In this study, the question asked is whether the AIR intervention enables timely home-discharge of bedridden tracheostomy patients, decreases complications, and reduces costs for patient-families as compared to those who do not have access to the intervention. People who are suitable and consent to take part will be divided into two groups based on having a tracheostomy. This is done to create two groups of patients, allowing for a more robust assessment of the effect of the AIR intervention. Quality of life of patients and Carer burden will be measured at discharge and on days after discharge using specially designed questionnaires. We will also collect information on OOPE of the patient-family during hospital stay and in the 1^st^ month after discharge, to understand if the AIR is cost-effective for the person and the health service. And finally, we will assess the thoughts, feedback, and live experiences of patient-carers who are enrolled in the study to identify barriers to wider implementation if we find that it benefits patient-carers and the healthcare service.

# 4.0 BACKGROUND AND RATIONALE

**4.1 Background**

The implications and outcomes (including cost) following protracted ICU stay are potentially devastating for patients who are tracheostomised and bedridden for chronic critical illness (CCI) and for their families, especially in lower-middle-income countries like India. Reasons for this include limited availability of and access to rehabilitation services, lower baseline health literacy, concomitant infections in the presence of antibiotic resistance, and spiralling financial burden associated with healthcare costs and loss of income.

For patients at risk of extended ICU stay and CCI, discharge home to be with family is a priority. Not only does timely discharge alleviate the burden of the cost of hospital care, but it may also reduce the incidence of HCAIs and provide families with opportunities to be at home with their loved ones as they attempt to recover. Structured interventions to enable family-led home rehabilitation of tracheostomised CCI patients are few and more often tailored for the paediatric population. (3) The barriers to implementation of family-led home rehabilitation of non-tracheostomised chronically ill adults are a lack of directed training, education, and support for family caregivers in the form of assistive devices and timely referral to health services. (4–8)

To meet this gap in Odisha, a state in Eastern India, a team of critical care physicians, nurses, rehabilitation specialists, and dieticians from the All-India Institute of Medical Sciences (AIIMS) designed an intervention to facilitate home rehabilitation of tracheostomised CCI patients. The endeavour was supported by the National Health Mission Odisha and Collaboration for Research, Training, and Implementation in Critical Care in Asia and Africa (CCAA)– a Wellcome Trust Innovations project.

The AIIMS ICU rehabilitation (AIR) project (Appendix 1) has designed an intervention to equip family caregivers to accept and execute home rehabilitation of chronically ill tracheostomised patients. It gives bedside education and training to carers for tracheostomy care, indwelling catheter care, nasogastric feeding, back care, suctioning, home ventilation management and troubleshooting. It has been pilot tested at a single centre and modified based on a realist review of international literature and stakeholder feedback for scale-up implementation at three centres.

This protocol describes a mixed-methods evaluation of the effectiveness and processes of the multicentric scaled-up implementation.

###### 4.2 Study Rationale

The AIIMS ICU Rehabilitation pilot project has recruited more than 200 tracheostomised chronically ill patients and their family carers for home rehabilitation. It has been pilot tested and has shown good feasibility and acceptability among stakeholders. Preliminary data show that it has reduced the length of ICU and hospital stays and frequency of hospital-acquired infections.

A realist review of international studies reporting interventions aiming to implement family-led home care of chronically ill patients indicates that interventions that include components of co-designed training, supported discharge, and home visits for post-discharge follow-up facilitate implementation and reduce the length of stay and rehospitalisation among chronically ill patients, such as stroke survivors.

# 5.0 STUDY DESIGN

###### 5.1 Controlled Interrupted time series design (work series 1)

A time series design is a continuous set of observations taken repeatedly over time. In this design, we will use a time series of our outcome of interest (time to hospital discharge) to establish an underlying trend that will be 'interrupted' by the AIR intervention at a known time. The expected trend in the absence of the intervention, or the counterfactual trend, will provide a comparator for evaluating the impact of the AIR intervention by interrogating the change in the post-intervention period. Fig. 2 illustrates the design with the graph displaying preintervention weekly trends of mean time to discharge, the counterfactual scenario (dashed line), and the hypothesised decrease in (mean) length of hospital stay every week. An appropriate ITS statistical model will be selected to provide evidence of whether the change represents a real decrease.



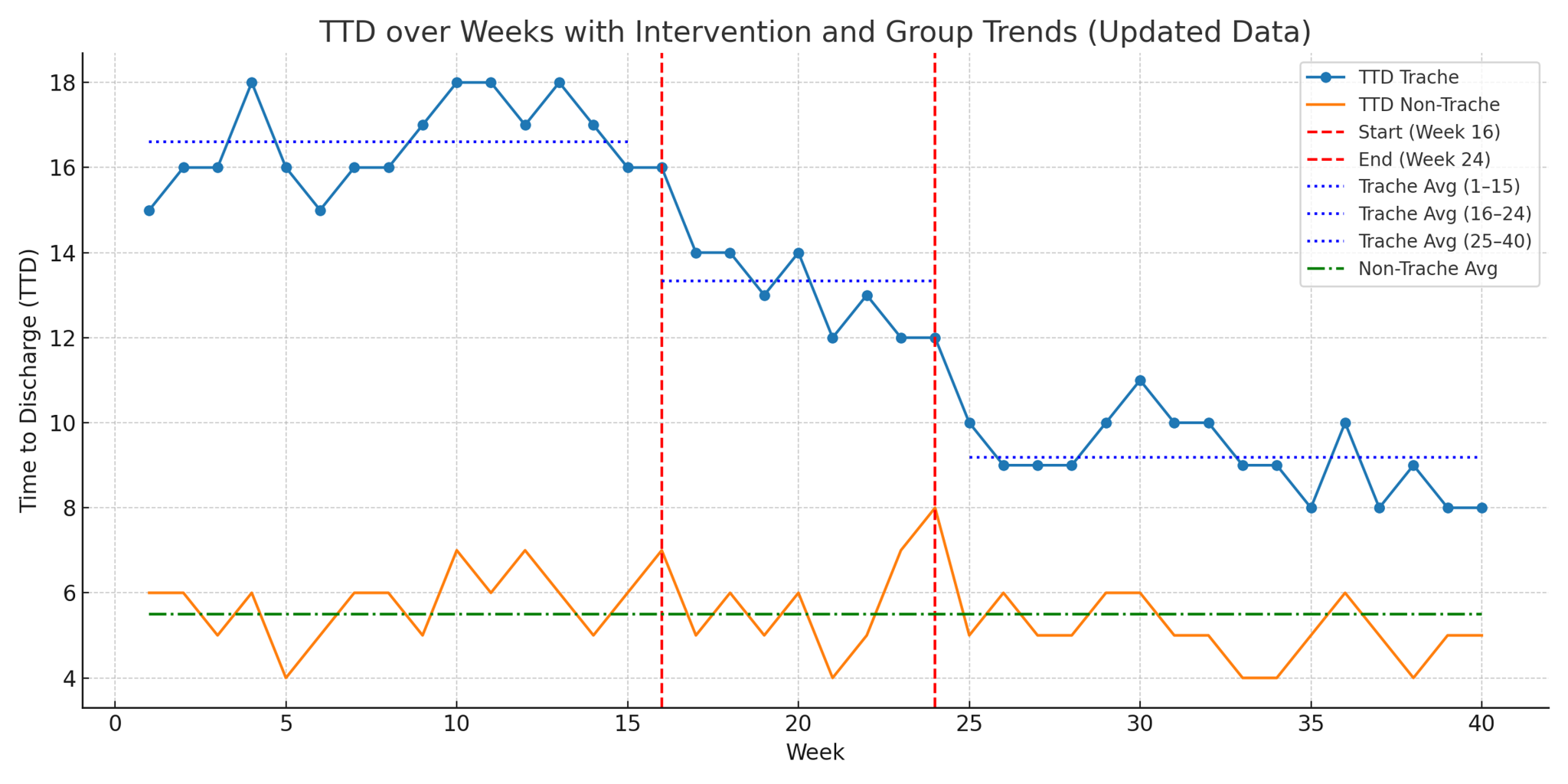


An ITS design is considered appropriate for our study for the following reasons-

1. An RCT is considered the gold standard for evaluating the effectiveness of interventions. However, where doing an RCT is not practical, such as in our setting, ITS design is increasingly used as it is particularly suited to interventions introduced over a clearly defined time period.
2. ITS works best with short-term outcomes expected to change quickly after an intervention is implemented. This is expected from the AIR intervention, which hopes to enable early supported discharge of patients.
3. ITS requires the availability of sequential measures of the outcome before and after the outcome, which will be possible in our study.
4. ITS is often used to evaluate natural experiments in real-world settings, thereby increasing external validity compared to RCTs.

The hypothesised impact model for our study is decided a priori based on literature and knowledge of the pilot expects a change in level without affecting the slope. If effective, the AIR intervention will follow the intervention immediately without a lag effect and decrease the average time to hospital discharge without affecting the slope (rate of discharge from the hospital), Fig. 1.

If the design of data collection within each time point does not lend itself to an ITS design, the data will be analysed using a pre-post comparison of time to discharge.

###### 5.2 Primary and secondary outcomes (TABLE 2)

**5.2.1 Primary outcome**

Time from the clinician declaring that the patient can be stepped down (Ready For Step Down) to the patient being discharged home.

**5.2.2 Secondary outcomes**

i. Patient and Carer Reported Outcomes measured at Day 30.

a. Health-related Quality of Life (QoL) with EuroQOL 3 dimensions (EQ-5D-3L) of patients at hospital discharge and at Day 30 after hospital discharge?

b. Carer Burden Scores (CBS) of carers at hospital discharge and at 30-day post-discharge

c. Alert, Verbal, Pain, Unresponsive (AVPU) scores at discharge and at 30-day

ii. Measures of safety

a. Hospital-acquired infections- central line-associated bloodstream infections, catheter-associated urinary tract infections, ventilator-associated pneumonia, surgical site infections, in the rate of events within groups per unit time.

b. Clinical outcomes – Length of stay in ICU, frequency of bed sores, tracheostomy blockage, Clinical Deterioration (as declared by the treating team in the hospital or deterioration in AVPU status at follow-up), readmission to ICU or hospital (after discharge and up to 30-day follow-up) and mortality at follow-up of 30 days (rate/ unit time).

iii. Measures of process (post-intervention stage)- Implementation Measures

1. Adherence to the intervention

- %Eligible patients approached
- % consented
- % Trained for at least 3 sessions
- Number of sessions missed.
- Subjective and objective scores of carers' confidence post-training
- % using the mHealth app at least once spontaneously after home discharge
- % 1 week home visits completed
- Data entry (completion)

| **Outcome** | **Definition** | **Assumed relationship** |
| --- | --- | --- |
| Time from the clinician's decision of readiness for step down | Time (days) from the Clinician declaring the patient ready for step down to discharge home under a carer or a paid caregiver.  *Home is defined as the usual residence, or family home, or a rental house in the vicinity of the hospital, but not another healthcare facility* | The intervention will reduce hospital length of stay/ bed days. |
| ICU/ HDU length of stay | Time from ICU admission to discharge to an intermediate facility (ie HDU/ ward) within the same hospital | The intervention will reduce ICU length of stay/ bed days. |
| Complications | Hospital-associated infections are defined as clinician-reported suspected/confirmed infection + new fever or new change in antibiotic. | There will be a reduction in HAI following the intervention. |
| 30 Day Outcomes | Mortality  Quality of Life (EQ5D)  Readmission to the same, or another healthcare facility  Caregiver Burden (by CBS scale) | There will be no worsening in patient and carer outcomes due to the intervention. |

*Table 2. Description of outcomes and assumed relationship with intervention.*

**5.2.3 Data Management**

All data will be collected through the Indian Registry of Intensive Care (IRIS) eCRF platform to ensure data safety and security; for the duration of the study, the site will be a part of the IRIS registry. Each site will have full rights over the data of non-study participants; only anonymised data will be shared with the registry.

**5.2.4 Recruitment and retention**

During the first 4 weeks of the recruitment, the feasibility of the timely completion of the study will be assessed using an internal pilot. Based on the pilot study, the targets will be:

1. Recruit a minimum of 2 tracheostomy and 2 non-tracheostomy patients per week per centre.
2. Participants drop out in the first 4 weeks, <25%.

###### 5.3 Embedded process evaluation and qualitative study (Workstreams 2 & 3)

The process evaluation examined usual care practices, intervention implementation, and maintenance across sites, with specific attention to fidelity, adaptation, and contextual determinants influencing adoption and delivery. During the pre-implementation phase, data focused on characterising usual care, discharge practices, and caregiver preparedness. During implementation and maintenance phases, observations and interviews were used to examine how intervention components were delivered, adapted, or not implemented, and the organisational and professional factors shaping these processes. Barriers and facilitators were explored iteratively using a realist-informed approach and subsequently mapped to the CFIR framework to support interpretation across sites and phases.

**5.3.1 Design and data collection**

A process evaluation will provide an assessment of the intervention's implementation, including how well the implementation follows the protocol (components, timing and duration) and identification of the contextual barriers and facilitators to adoption and implementation. A mixed methods approach will be used whereby quantitative data (as collected from Workstream 1) and qualitative data in the form of field notes, observations, interviews and discussions with the implementation team will be analysed.

**Data sources and types, and timepoints**

**Field notes**

1a. Will be generated during the pre-implementation phase at all sites to examine interpretation, acceptability, and contextual readiness, informed by more than one visit by the central research team and site research personnel;

1b. Three team stakeholder meetings at each site conducted at baseline(pre implementation), at the start of implementation, and during the maintenance phase.

**Observations.**

2. Non-participant observations undertaken by the research team will be taken throughout the study , documenting patient- family- clinician interactions and care processes relevant to the implementation and intervention.

**Semi- structured interviews**

3. Semi-structured interviews with patient–carer dyads conducted approximately one month after recruitment (recruitment is at the time of RFSD), including 3a. Carers who received the intervention and

3b. baseline-period participants describing their lived experience of home discharge without the intervention;

3c. Semi-structured interviews with ICU nursing staff and Site PIs exploring perceptions of usual care, experiences of the intervention, and expectations for future home discharge will be undertaken after completion of the study

**Debriefs**

4. Individual debriefs and group reflective discussions with the project team conducted throughout the study period. (looking at usual care, experiences and expectations).

The study will aim to recruit approximately 12 patient-carers, 6 healthcare workers, and 4 project staff, with sampling targets reviewed iteratively alongside concurrent analysis.

**5.3.2 Recruitment, and consent**

The AIRe study team and researcher undertaking the process evaluation will liaise with the site PI and unit manager to identify suitable observation periods. Before the study begins, the lead researcher will visit each site and explain the process evaluation, allowing staff to ask questions or express concerns.

*a) Observations*

The patient-family of the non-control group being observed will be formally recruited and fully informed about the study. Written consent will be obtained before undertaking observations. For the healthcare workers, formal recruitment will not be practised, but they will be informed about the study, the processes, and their roles. They will be free to deny participating in observations . The researcher will obtain verbal permission to conduct observations on the unit overall. The researcher will ask the ward manager to circulate an email to staff to inform them before observations take place, along with a copy of the participant information sheet. Permissions and refusals will be documented by the researcher, and each observation will last approximately 6-7 hours.

*b) Interviews*

Eligible HCWs/patient-family dyads will be invited by a member of the clinical and/ or the research team to engage in a semi-structured interview. A time and situation conducive to interviews will be identified in every centre, but the site selected may be changed to suit the work pressures and business of the interviewees. Where the interviews are at patient homes (telephonic or physical), at least 24 hrs will be given to suggest a suitable date and time for the meeting. Withdrawal from the study is allowed within the 4 weeks following the interview. The researcher will identify and destroy the data if consent is withdrawn. This will be recorded.

**5.3.3Process evaluation analysis**

Transcripts of all data types (observations, field notes and interviews) will be deductively analysed independently by two researchers (SS ST) to identify the key concepts (themes) regarding intervention acceptability, along with barriers and facilitators to implementation. Initially the text will be open-coded by reviewing all text line by line and then descriptive codes will be assigned to the words, sentences and paragraphs in the transcripts. At this stage of the analysis, lines in the transcripts will be linked and grouped as the first set of codes which relate to the current prescribing practices, the experiences of the patient and family dyads, the clinical teams, and research team, along with adaptations to intervention and implementation made. Axial coding in the next step of the data reduction process will link the descriptive codes via repackaging and combine the data to identify categories that have similar characteristics. After developing categories, the relationships between the categories will be explored to reveal higher level themes, and then where relevant to implementation of the intervention mapped to the CFIR framework 2. New themes, and contextual and team factors that emerge from the evaluation but do not fit the existing framework will also be reported.

#

#

# 6.0 PARTICIPANT ELIGIBILITY CRITERIA

###### 6.1 Inclusion Criteria for Controlled Interrupted Time Series (Workstream 1)

1. Adult tracheostomised patients in the neurosurgery ICU, expected to survive beyond and need tracheostomy for more than 2 weeks after being declared 'expected fit to discharge' from the ICU by the treating physician/ surgeon.

2. Age ≥ 18 years

3. Ability and willingness to give written informed consent by Dyads..

###### 6.2 Exclusion Criteria for Controlled Interrupted Time Series (Workstream 1)

1. Patients without an identified family carer willing for home care,

2. Patients needing greater organ support than is manageable at home (home ventilation excluded)

Current participation in an interventional trial with conflicting therapies or primary outcomes

3. Staying beyond a 100 km radius, precluding scheduled home visit

The inclusion and exclusion criteria are broad-based and pragmatic and therefore maximise external validity of the study. The primary outcome is chosen to maximise inclusion of all eligible patients who can safely be cared for at home. Short patient life expectancy post-discharge has not been considered a criterion for exclusion, with the understanding that this will enable informed decision and safe end-of-life care of these patients, often a reality in neuro ICUs. External factors that may influence the primary outcome, such as contextual barriers to the clinician declaring fit-for-discharge or the families' reluctance for home care, have been included to address potential sources of bias arising from them.

###### 6.3 Additional eligibility criteria for process evaluation (Workstream 2)

**6.3.1 Staff participant inclusion criteria**

1. Clinical and non-clinical staff working at the ICU and wards participating in the AIRe study.
2. Age ≥ 18 years
3. Ability to give written informed consent.
4. Ability and willingness to participate in the observations and interviews.

**6.3.2 Staff participant exclusion criteria**

1. Age <18 years
2. Unable to give written informed consent.
3. Unable or unwilling to participate in the observations and interviews.

**6.3.3 Relative/visitor participant inclusion criteria**

1. Relatives accompanying patients (who fulfil workstream one eligibility criteria)
2. Age ≥ 18 years
3. Ability to give written informed consent.
4. Ability and willingness to participate fully in the observations and interviews.

**6.3.4 Relative/visitor participant exclusion criteria**

1. Relatives accompanying patients who do not fulfil the eligibility criteria.
2. Age <18 years
3. Unable or unwilling to participate in the observations and interviews.

###### 6.4 Additional eligibility criteria for qualitative study (Workstream 3)

**6.4.1 Staff participant inclusion criteria**

1. Stakeholders (healthcare workers, project staff, hospital administrative staff) involved in overseeing or recruiting to the AIRe study.
2. Age ≥ 18 years
3. Ability to give informed consent.

**6.4.2 Staff participant exclusion criteria**

1. Age <18 years
2. Unable to give informed consent.

# 7.0 STUDY LOCATION

The study will be located at three tertiary care hospitals in Odisha, India. After home discharge, patient-carer dyads will be followed at home for 30 days over the phone, via a mHealth application and a home visit.

# 8.0 DURATION OF ENROLLMENT AND FOLLOW-UP

The trial will begin pre-intervention data collection for tracheostomised patients for 4 months before implementation of the intervention (over 30 days). After the implementation, data will be collected for 4 months. The individual participant will be enrolled in the ICU and followed up after home discharge for 30 days.

# 9.0 DESCRIPTION OF INTERVENTION AND ADMINISTRATION

The processes of intervention administration will broadly follow those of the pilot intervention.

(Fig. 3 & 4)

###### 9.1 Schedule of Evaluations

|  | ***Screening*** | ***Consent & Rapport Building*** | ***Training D1-D4*** | ***Pre Discharge*** | ***Post Discharge D1-3*** | ***Post Discharge D 6-7*** | ***Post Discharge D28-31*** | ***Follow up*** |
| --- | --- | --- | --- | --- | --- | --- | --- | --- |
| Informed Consent Form | ***X*** |  |  |  |  |  |  |  |
| LIAISE with Treating Team | ***X*** |  |  |  |  |  |  |  |
| Demographics | ***X*** | ***X*** |  |  |  |  |  |  |
| Primary assessment | ***X*** |  |  |  |  |  |  |  |
| Training Assessment |  |  | ***X*** |  |  |  |  |  |
| In hospital/post-discharge  Hai/ new infections  ICU/hospital readmission  Mortality  Adverse events  Cost  QOL, CBS |  | ***X***  ***X***  ***X***  ***X*** | ***X***  ***X***  ***X***  ***X***  ***X*** | ***X***  ***X***  ***X***  ***X***  ***X***  ***X*** | ***X***  ***X*** | ***X***  ***X***  ***X*** | ***X***  ***X***  ***X***  ***X***  ***X***  ***X*** |  |
| Pre-discharge assessment |  |  |  | ***X*** |  |  |  |  |
| mHealth survey |  | ***X*** | ***X*** | ***X*** |  |  | ***X*** |  |
| Day 1-2-3 assessment |  |  |  |  | ***X*** |  |  |  |
| Day 7 assessment |  |  |  |  |  | ***X*** |  |  |
| Day 28 assessment |  |  |  |  |  |  | ***X*** |  |
| General Assessment Form |  |  |  |  |  |  |  | ***X*** |

###### *Table 2: Schedule of evaluations in the intervention group.*

**9.2 Description of Evaluations:** Detailed description in the manual of the intervention and evaluations

###### 9.3 Adverse Events and Serious Adverse Events

***Serious Adverse Event (SAE):*** Any adverse event that:

- Results in death
- Is it threatening, or places the participant at immediate risk of death from the event as it occurred?
- Requires or prolongs hospitalisation.
- Causes persistent or significant disability or incapacity.

# 10. DATA COLLECTION TIME POINTS

| **Timepoint** | **Grouped Variables Collected (Indicative Examples)** | **T pi** | **NT** | **C** |
| --- | --- | --- | --- | --- |
| Recruitment | Demographics: Name, Age, Sex, MRN, Contact No.  Administrative: Fee status, Unit/Hospital Admission/Discharge dates and times  Clinical: Comorbidities, Admission source/type/reason, SARI  Tracheostomy-specific: Tracheostomy date & reason, Previous inclusion  Outcomes: Nature of disposal | ✔ | ✔ p | ✔ p |
| Post Enrollment | Vitals & Parameters: RR, HR, BP, Temp, GCS, AVPU, SpO2, PaO2, PaCO2, FiO2, Arterial pH  Labs: Hb, WBC, Platelets, Electrolytes (Na+, K+, HCO3-), Creatinine, Bilirubin, PCV, Urea  Support: CV support, Vasopressors, Sedation, Renal replacement  Treatment: Antimicrobial use & type  Functional: Clinical frailty, Patient status/location  Trache-specific: Seizures, Oxygen flow rate | ✔ | ✔p | ✔ |
| In ICU (Daily) | Daily Vitals: RR, HR, BP, Temp, GCS, SpO2, FiO2, PaO2  Support: CV support, Vasopressors, Sedation  Access: CVC, arterial line, urine catheter, HD catheter  Imaging & Labs: Lung radiology, Platelets, WBC (high/low), Creatinine, Bilirubin, PT  Treatment: Antimicrobials, Specimen collection/report  Safety: Pressure sores  Trache-specific: Oxygen flow, Seizures, Location | ✔ | ✔ p | ✔ |
| Post ICU (Up to Discharge) | RFSD Day Status: Patient/location status, date, timepoint  Socio-Demographics: Education, occupation, income, SES, eHealth/digital literacy (patient & caregiver)  CG Details: Name, age, gender, relation, contact, address  Clinical: Devices (PEG/NG/Jejunostomy, tracheostomy tube), vasopressors, renal replacement, stepped-down status  Outcome Monitoring: EQ5D, VAS, Health issues, Interventions | ✔ | ✘ | ✘ |
| During Training | Trainee Info: Name, age, gender, relationship, education, contact  Scores: Pre/post training scores (suction, feeding, bed care), Trainer observations  FAQs Topics: Tracheostomy, suctioning, feeding, hygiene, diet, catheter care, general questions | ✔ | ✘ | ✘ |
| Exit Training | Exit Training: Date, final training score | ✔ | ✘ | ✘ |
| Mobile App Distribution | Logistics: Days since RFSD, time taken  User Feedback: Issues, facilitators/barriers, app utility for training | ✔ | ✘ | ✘ |
| Equipment Delivery | Equipment Provided: Type, provider, training demo done  Logistics: Delay, placement guidance, agreement to T&C, ergonomics discussion  Barriers and facilitators | ✔ | ✘ | ✘ |
| Home Visit | Care Quality: Hygiene, equipment use, trache/RT/foley care, calorie tracking, mobilisation  Support: App usage, local HCC contact, medications | ✔ | ✘ | ✘ |
| At Discharge | Dates & Status: Unit/hospital discharge date & time, CPR, discharge note  Trache-Specific: Ongoing trach care, reason for early discharge, diet chart  CG Consistency: Same carer since enrollment, eHealth tools & scores  Outcome Measures: EQ5D, VAS, CBS score, Semi-structured interview | ✔ | ✔ p | ✔ |

Table 3. Data to be collected at different time points. Tpi-Tracheostomy (post-implementation), NT=Non-Tracheostomy, C= pre implementation patients with Tracheostomy) , p=partial

# 11. AMENDMENTS TO INITIAL PLAN AND PROTOCOL WITHIN THE STUDY PERIOD

**11.1 Rationale**

During the implementation of the AIR intervention scale-up, several amendments were necessitated based on the concurrent process evaluation findings from the three selected centres. The initial plan involved implementing the family-supported home discharge model for chronically ill tracheostomised patients RFSD at three new centres after it had been discontinued at the pilot site, AIIMS Bhubaneswar, and conducting process evaluations at each site to assess feasibility, fidelity, and contextual adaptability.

**Description of Change**

Findings from the process evaluation revealed substantial inter-site variability in context and stakeholder engagement.

- **Site A**, a tertiary teaching hospital, demonstrated high motivation among two key neurosurgeons and a neuroanesthesiologist intensivist. However, despite their support, a key barrier was the reluctance to discharge tracheostomised patients expected to recover and be decannulated within a few weeks, potentially influencing the primary outcome.
- **Site B**, also a medical teaching institution but with a higher patient load and lower socioeconomic profile, had a cohesive neurosurgical and ICU team already practising informal family task-sharing. Their main concern related to ensuring the safety and consistency of family-led care amidst high turnover.
- **Site C**, a private tertiary hospital serving higher socioeconomic groups, had interested intensivists but limited support from the neurosurgical team, possibly due to conflicts of interest linked to ownership of private post-discharge facilities. While baseline data collection was completed, implementation was actively hindered by the neurosurgical unit and affiliated post-discharge providers, making intervention delivery unfeasible.

In view of these barriers, after four months of baseline data collection and four weeks of attempted implementation, Site C was withdrawn from the implementation. To preserve comparability across three sites and optimise use of staff and resources, the AIR intervention was reinstated at the original pilot site—AIIMS Bhubaneswar (Site D). Since the program infrastructure had been discontinued earlier, a stakeholder re-engagement meeting was held with neurosurgery, neurology, trauma, and ICU teams to reintroduce the intervention. A four-month baseline data collection period preceded reimplementation to avoid contamination as far as possible. Stakeholders were informed of the reintroduction only after baseline data collection was completed. Figure 2.


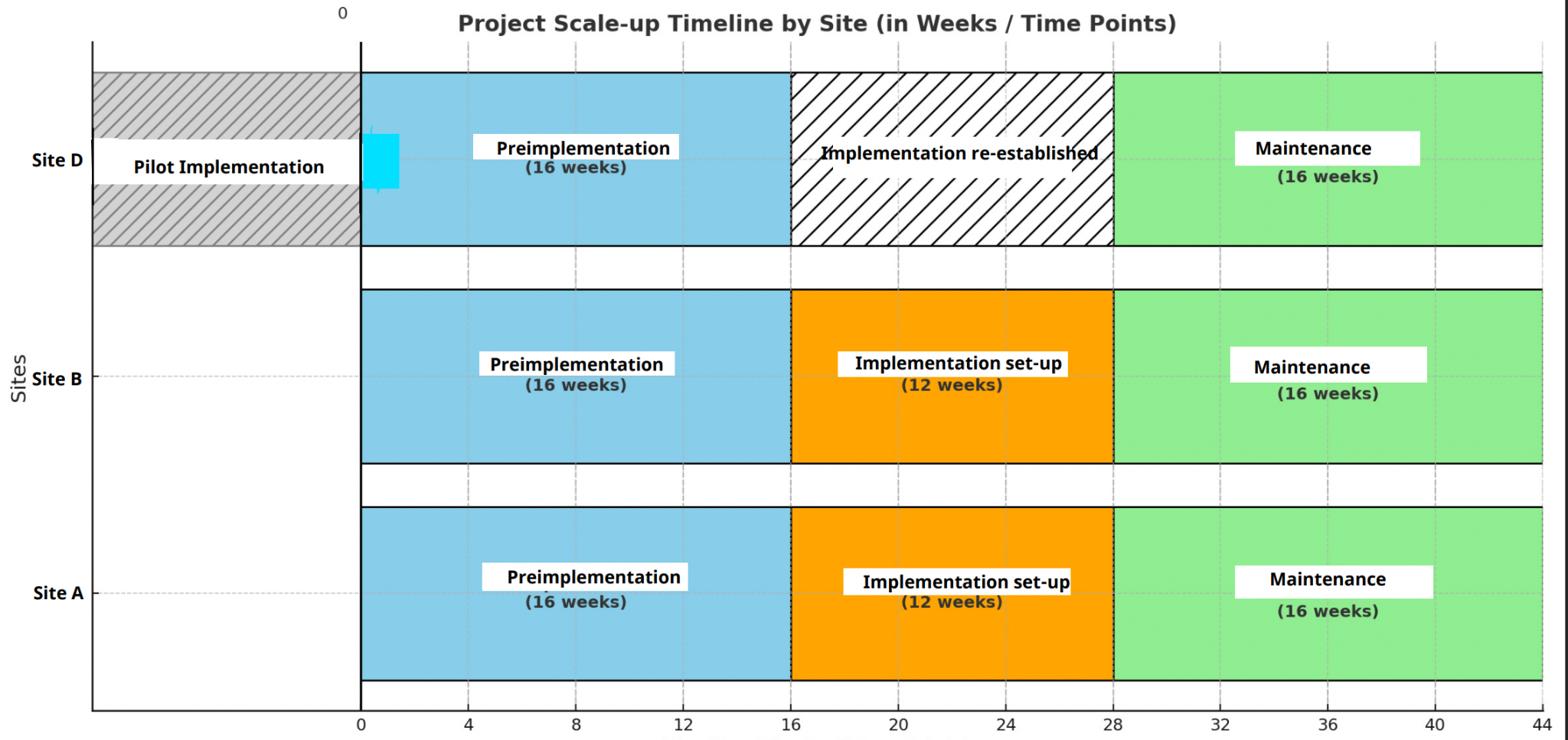


Figure 2. Project Timelines. Pre-implementation (blue) = usual care and baseline observation; Implementation (orange) = active intervention delivery; Maintenance (green) = sustained delivery under routine conditions; Diagonal hatching = non-routine phases (interrupted pilot activity or re-established implementation).Site C had 16 weeks of Pre-Implementation and 4 weeks of Implementation phases, and is not displayed here.

Note-Time is shown in study weeks (analytic time points) and is not calendar-aligned; phase transitions reflect site-specific initiation and interruption rather than fixed dates.

**Amendment to Statistical Analysis Plan**

As per the initial protocol, the analysis plan proposed conducting either an **interrupted time series (ITS)** analysis or a **pre–post design**, contingent on the achieved sample size. However, following the withdrawal of Site C, it was decided that the **primary analysis** would be a **pre–post comparison** of outcomes with Sites A and B, assessing differences before and after intervention implementation. In addition, a **secondary interrupted time series analysis** would be conducted, incorporating data from Sites A, B, and D (AIIMS Bhubaneswar) to evaluate temporal trends across these three participating centres. This modification ensured analytical rigour and retained the study's capacity to assess both immediate and longitudinal impacts of the AIR intervention.

The process evaluation was amended to align the planned methods with how implementation unfolded in practice across sites. While the initial plan assumed a uniform, time-bounded process evaluation across participating centres, early fieldwork identified inter-site variation in organisational readiness, discharge norms, and stakeholder engagement that directly influenced intervention delivery. Accordingly, the process evaluation was refined to emphasise longitudinal, observation-led inquiry across pre-implementation, implementation, and maintenance phases; to expand the use of ethnographic data and iterative analysis; and to explicitly examine contextual determinants of adoption, adaptation, and non-implementation. Analytical methods were clarified to include inductive realist-informed thematic analysis with subsequent deductive application of the CFIR framework. These amendments ensure that the process evaluation accurately captures fidelity, adaptation, and contextual constraints affecting delivery and supports valid interpretation of effectiveness outcomes

**Impact on Study Conduct**

These amendments were implemented to maintain scientific validity, optimise resource use, and adapt to real-world contextual challenges while preserving fidelity to the intervention's core objectives. All changes were documented, reviewed by the supervisory team, and communicated to stakeholders at each participating site.

# 12.0 REFERENCES

1. de Franca SA, Tavares WM, Salinet ASM, Paiva WS, Teixeira MJ. Early Tracheostomy in Severe Traumatic Brain Injury Patients: A Meta-Analysis and Comparison With Late Tracheostomy. Crit Care Med. 2020;48(4):e325–31.

2. Craig P, Dieooe P, Macintyre S, Michie S, Nazareth I, Petticrew M. Developing and evaluating complex interventions: Following considerable development in the field since 2006, MRC and NIHR have jointly commissioned an update of this guidance to be published in 2019. Medical Research Council [Internet]. 2019;1–39. Available from: https://mrc.ukri.org/documents/pdf/complex-interventions-guidance/

3. Oberwaldner B, Eber E. Tracheostomy care in the home. Paediatr Respir Rev. 2006 Sep;7(3):185–90.

4. Daraie S, Hasanvand S, Goudarzi F, Rassouli M. Gaining Experience Over Time: The Family Caregivers' Perception of Patients with a Tracheostomy in Home Care. Iran J Nurs Midwifery Res [Internet]. 2021 [cited 2022 Feb 15];26(2):137. Available from: /pmc/articles/PMC8132866/

5. Silver HJ, Wellman NS, Galindo-Ciocon D, Johnson P. Family caregivers of older adults on home enteral nutrition have multiple unmet task-related training needs and low overall preparedness for caregiving. J Am Diet Assoc. 2004;104(1):43–50.

6. SS Kun SDWLHTK. How much do primary care givers know about tracheostomy and home ventilator emergency care? Pediatr Pulmonol. 2010 Mar;45(3):270–4.

7. Udchumpisai M. Thriving in Family Caregiving: A Grounded Theory Study of Thai Family Caregivers of Patients with Home Mechanical Ventilation (HMV).

8. Bains P, Minhas AS. Profile of home-based caregivers of bedridden patients in North India. Indian Journal of Community Medicine. 2011;36(2):114–9.


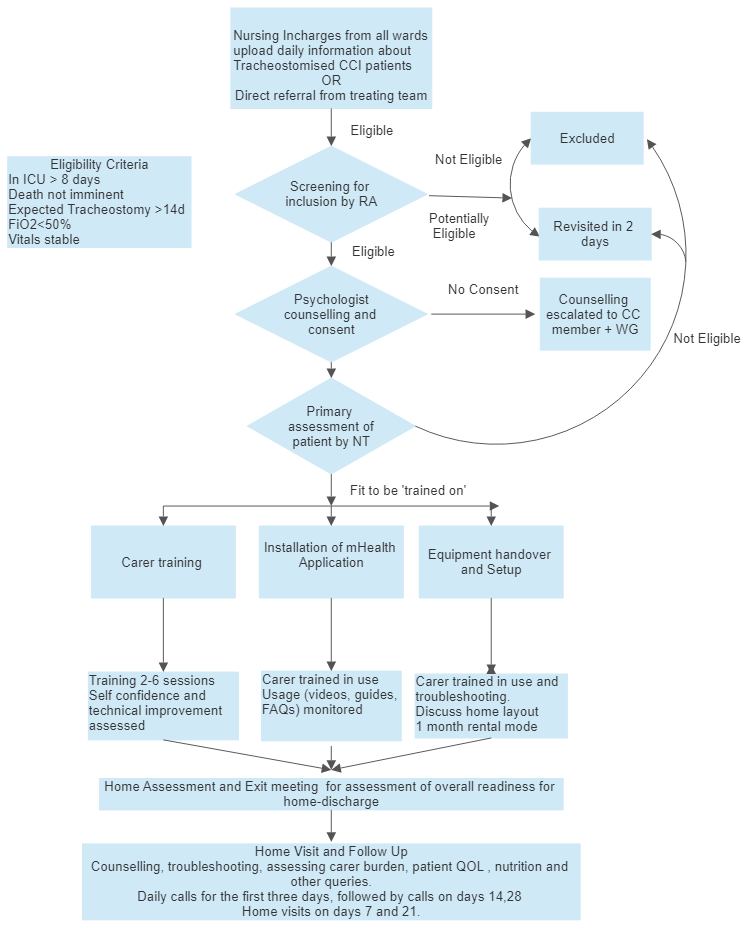


Fig 3. Processes and procedures used in the intervention

RA Research Assistant, CCI Chronically Critically Ill, WG Working Group ( including the Treating doctor), CC Core Committee, NT Nurse Trainer, QOL- Quality of Life


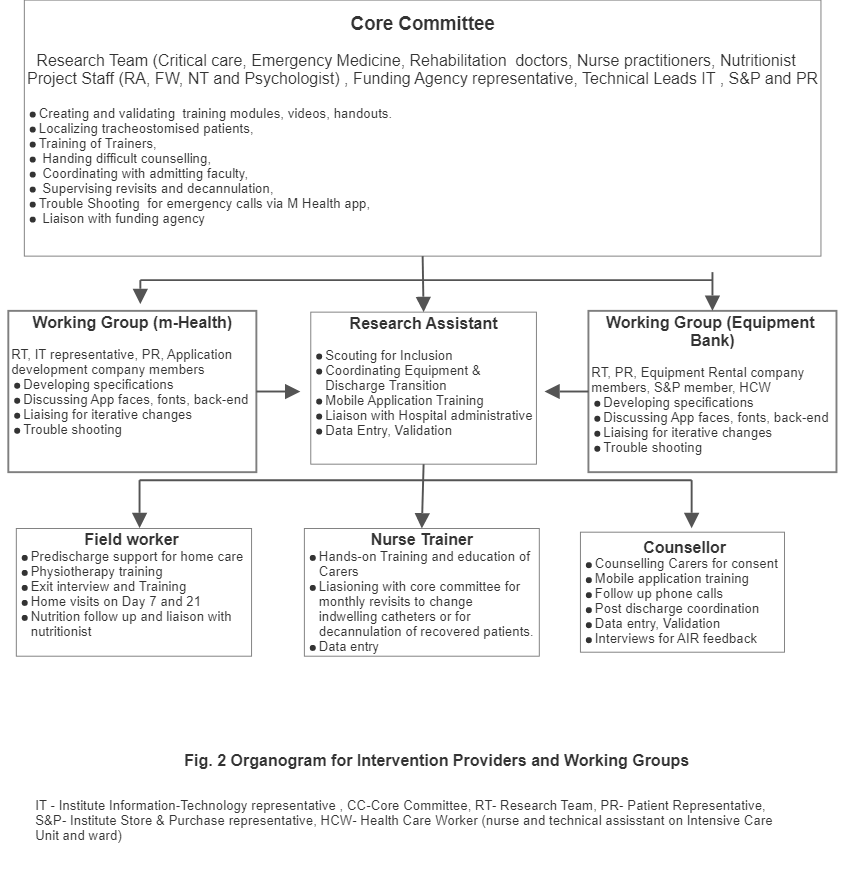

Supplement: S1 File — (DOCX) [file pone.0348345.s005.docx]
