# Supplementary material for: Family-led post-ICU discharge intervention for tracheostomized patients in India: Feasibility and formative impact evaluation
Source: PLoS One. 2026 May 29;21(5):e0348345. doi: 10.1371/journal.pone.0348345 (PMC13221049; doi:10.1371/journal.pone.0348345)
Supplement: S2 File — (DOCX) [file pone.0348345.s006.docx]

## Supplement 7. Semi‑Structured Interview Guide for Stakeholder Interviews

This guide was used to explore perceived barriers and facilitators to implementation of the AIR intervention among caregivers and healthcare stakeholders. Questions served as prompts and interviewers adapted wording and sequence depending on participant responses.

| Domain | Initial Question | Possible Probing Prompts |
| --- | --- | --- |
| Caregiving Experience | Can you describe your experience caring for your family member after ICU discharge? | Time spent caring; support from family members; major challenges encountered; emotional or financial impact. |
| Transition from Hospital to Home | Can you tell me about the process of bringing your family member home from hospital? | Factors influencing the decision; expectations from hospital staff; preparation for home care; concerns at discharge. |
| Training and Preparation | What did you think about the caregiver training provided? | Most useful aspects of training; areas needing improvement; confidence in performing care tasks; repetition or reinforcement of skills. |
| Equipment Support | How helpful was the equipment provided or recommended for home care? | Most frequently used devices; difficulties using equipment; affordability and availability; suggestions for improving access. |
| Digital Communication and Support | Did you use mobile communication or digital resources to support caregiving? | Experience with the AIR mobile app; use of WhatsApp or phone communication; barriers such as internet connectivity or device availability. |
| Post‑Discharge Follow‑up | What support did you receive after returning home? | Telephone calls; home visits; troubleshooting advice; perceived importance of follow‑up support. |
| Perceived Benefits and Challenges | What aspects of the AIR programme were most helpful? | Impact on caregiver confidence; perceived improvements in patient care; barriers to participation; suggestions for improving the programme. |
| Concluding Questions | Is there anything else you would like to share about your experience? | Additional suggestions for improving caregiver support or hospital discharge preparation. |
