# Supplementary material for: Family-led post-ICU discharge intervention for tracheostomized patients in India: Feasibility and formative impact evaluation
Source: PLoS One. 2026 May 29;21(5):e0348345. doi: 10.1371/journal.pone.0348345 (PMC13221049; doi:10.1371/journal.pone.0348345)
Supplement: Attachment — Submitted filename: Response to Editorial Office PLOS.docx [file pone.0348345.s007.docx]

**Response to Editorial Office – PONE-D-25-65437**

Dear Editorial Team,

Thank you for the opportunity to revise our submission. We have now addressed all points raised in the editorial office letter and confirm that the requested documents and revisions have been completed and uploaded. Please find our responses below.

1. **Clinical trial registration**
   In line with PLOS ONE requirements, we confirm that this study meets the WHO definition of a clinical trial. The trial has been prospectively registered with the **Clinical Trials Registry of India (CTRI)**. The registry name and registration number have now been included in the submission metadata and on the manuscript title page.
   The complete date range for participant recruitment and follow-up has been added to the Methods section.
2. **CONSORT / TREND checklist**
   As this is a non-randomised, formative implementation study, we have completed and uploaded the **TREND checklist** as Supporting Information, in accordance with journal guidance.
3. **Study protocol upload**
   We have uploaded the **original IEC-approved study protocol** as Supporting Information.
   Minor clarificatory language has been embedded in the protocol to explicitly reflect the pilot, formative, and adaptive nature of the intervention, without altering the original intent, ethical scope, or risk profile.
   Any adaptations undertaken during implementation are transparently described in the Methods section of the manuscript.
4. **Ethics approval documentation**
   We have now uploaded:
   - The **initial IEC approval letter**, and this allows permission till the end of study.
5. **Manuscript revisions**
   All corresponding edits have been incorporated into the revised manuscript, and all requested supporting documents have been uploaded. The revised PDF has been checked and approved.

We trust that these revisions fully address the editorial requirements and thank the editorial team for their careful review. We would be pleased to provide any further clarification if needed.

Kind regards,
**Prof. Swagata Tripathy**
(on behalf of all authors)
